# New Genome Sequence of an *Echinacea* *purpurea* Endophyte, *Arthrobacter* sp. Strain EpSL27, Able To Inhibit Human-Opportunistic Pathogens

**DOI:** 10.1128/genomeA.00565-17

**Published:** 2017-06-22

**Authors:** Elisangela Miceli, Luana Presta, Valentina Maggini, Marco Fondi, Emanuele Bosi, Carolina Chiellini, Camilla Fagorzi, Patrizia Bogani, Vincenzo Di Pilato, Gian Maria Rossolini, Alessio Mengoni, Fabio Firenzuoli, Elena Perrin, Renato Fani

**Affiliations:** a Department of Biology, University of Florence, Florence, Italy; b Department of Experimental and Clinical Medicine, University of Florence, Florence, Italy; c Center for Integrative Medicine, Careggi University Hospital, University of Florence, Florence, Italy; d Department of Surgery and Translational Medicine, University of Florence, Florence, Italy; e Clinical Microbiology and Virology Unit, Careggi University Hospital, Florence, Italy

## Abstract

We announce here the draft genome sequence of *Arthrobacter* sp. strain EpSL27, isolated from the stem and leaves of the medicinal plant *Echinacea purpurea* and able to inhibit human-pathogenic bacterial strains. The genome sequencing of this strain may lead to the identification of genes involved in the production of antimicrobial molecules.

## GENOME ANNOUNCEMENT

Medicinal plants are well known and have been largely explored for centuries for their therapeutic properties (1). What is little known is that their therapeutic potential could be related to endophytic microorganisms inhabiting their tissues (2). Many bioactive molecules have been already extracted from endophytic bacteria (3). The promising potential of such organisms has led to the characterization of endophytic bacterial communities from medicinal plants, which are poorly known. Endophytic and rhizospheric bacterial communities from the medicinal plants *Echinacea purpurea* and *Echinacea angustifolia* have been characterized, highlighting the specific composition of such communities within plants’ compartments (4). *Arthrobacter* sp. strain EpSL27, extracted from the stem and leaves of *E. purpurea*, has been evidenced as being resistant to a high level of oxidative stress (20 mM H_2_O_2_) and is able to degrade diesel fuel. Among such notable biotechnological potentialities, *Arthrobacter* sp. EpSL27 has also been found to show strong inhibition activity toward human-pathogenic bacteria from the *Burkholderia cepacia* complex (5), which are multidrug-resistant organisms able to induce serious infections in immunocompromised patients.

The intriguing information obtained by the above-cited analyses led to whole sequencing of the strain genome.

*Arthrobacter* sp. EpSL27 genomic DNA was extracted using the cetyltrimethylammonium bromide (CTAB) method (6), and its authenticity has been confirmed by 16S rRNA gene sequencing. Whole-genome shotgun sequencing was performed with a 2 × 300-bp paired-end approach using the MiSeq sequencing system (Illumina, Inc., San Diego, CA). The FastQC software package version 0.52 (7) was used to evaluate the quality of the obtained read pairs, and poor-quality bases were removed using StreamingTrim (8). Assembly was performed using the SPAdes 3.5 software (9), with k-mer lengths of 21, 33, and 55, generating 21 contigs. Those having a length shorter than 200 nucleotides were removed and the others launched for scaffolding through Medusa software (10), using the following genomes as references: *Arthrobacter arilaitensis* Re117 (11), *Arthrobacter* Rue61a (12), *Arthrobacter* sp. strain FB24 (13), *Arthrobacter aurescens* TC1 (14), and *Arthrobacter chlorophenolicus* A6. The resulting scaffolds were then annotated using the NCBI Prokaryotic Genome Annotation Pipeline (PGAAP) (15). The final version of the *Arthrobacter* sp. EpSL27 draft genome consists of 8 scaffolds, and its total length is 4,176,054 bp, with a coverage of 215.0×. The G+C content is about 67.8%, which reflects the characteristic high G+C content of the genus. The *Arthrobacter* sp. EpSL27 genome harbors 3,758 genes, 3,610 of which are protein-coding genes, 66 are RNA-coding genes (5 5S rRNA, 1 23S rRNA, 1 16S rRNA, 50 tRNAs, and 9 noncoding RNA [ncRNA]), and 91 are pseudogenes.

The EpSL27 genome was analyzed using CARD (16) for the presence of genes conferring antibiotic resistance. The analysis has evidenced genes putatively involved in specific antibiotic resistance to isoniazid (*Mycobacterium tuberculosis kasA* mutant), fluoroquinolones (*mfd*), amynocoumarin (*Streptomyces rishiriensis parY* mutant), rifamycin (*rphB*), mupirocin (*Bifidobacterium* intrinsic *ileS*), and fosfomycin (*Chlamydia trachlomatis* intrinsic *murA*). antiSMASH (17) analysis for secondary metabolites with antimicrobial activities was also performed, revealing the presence of 5 clusters, with one cluster encoding nonribosomal peptide synthetase (NRPS), one cluster encoding type 3 polyketide synthase (T3pks), and another three clusters with an unspecified reference.

### Accession number(s).

The whole-genome shotgun project has been deposited at NCBI whole-genome sequencing (WGS) database under accession number LNUT00000000, and the version reported in this work is version LNUT00000000.1.

## References

[1] Staub PO, Casu L, Leonti M 2016 Back to the roots: a quantitative survey of herbal drugs in Dioscorides’ de materia medica (ex Matthioli, 1568). Phytomedicine 23:1043–1052. doi:10.1016/j.phymed.2016.06.016. 27444350

[2] Ryan RP, Germaine K, Franks A, Ryan DJ, Dowling DN 2008 Bacterial endophytes: recent developments and applications. FEMS Microbiol Lett 278:1–9. doi:10.1111/j.1574-6968.2007.00918.x. 18034833

[3] Shweta S, Bindu JH, Raghu J, Suma HK, Manjunatha BL, Kumara PM, Ravikanth G, Nataraja KN, Ganeshaiah KN, Uma Shaanker R 2013 Isolation of endophytic bacteria producing the anti-cancer alkaloid camptothecin from *Miquelia* *dentata* Bedd. (Icacinaceae). Phytomedicine 20:913–917. doi:10.1016/j.phymed.2013.04.004. 23694750

[4] Chiellini C, Maida I, Emiliani G, Mengoni A, Mocali S, Fabiani A, Biffi S, Maggini V, Gori L, Vannacci A, Gallo E, Firenzuoli F, Fani R 2014 Endophytic and rhizospheric bacterial communities isolated from the medicinal plants *Echinacea* *purpurea* and *Echinacea* *angustifolia*. Int Microbiol 17:165–174. doi:10.2436/20.1501.01.219. 26419456

[5] Chiellini C, Maida I, Maggini V, Bosi E, Mocali S, Emiliani G, Perrin E, Firenzuoli F, Mengoni A, Fani R 2017 Preliminary data on antibacterial activity of *Echinacea* *purpurea*-associated bacterial communities against *Burkholderia cepacia* complex strains, opportunistic pathogens of cystic fibrosis patients. Microbiol Res 196:34–43. doi:10.1016/j.micres.2016.12.001. 28164789

[6] Perrin E, Fondi M, Maida I, Mengoni A, Chiellini C, Mocali S, Cocchi P, Campana S, Taccetti G, Vaneechoutte M, Fani R 2015 Genomes analysis and bacteria identification: the use of overlapping genes as molecular markers. J Microbiol Methods 117:108–112. doi:10.1016/j.mimet.2015.07.025. 26235543

[7] Kunde-Ramamoorthy G, Coarfa C, Laritsky E, Kessler NJ, Harris RA, Xu M, Chen R, Shen L, Milosavljevic A, Waterland RA 2014 Comparison and quantitative verification of mapping algorithms for whole-genome bisulfite sequencing. Nucleic Acids Res 42:e43. doi:10.1093/nar/gkt1325. 24391148 PMC3973287

[8] Bacci G, Bazzicalupo M, Benedetti A, Mengoni A 2014 StreamingTrim 1.0: a Java software for dynamic trimming of 16S rRNA sequence data from metagenetic studies. Mol Ecol Resour 14:426–434. doi:10.1111/1755-0998.12187. 24128146

[9] Bankevich A, Nurk S, Antipov D, Gurevich AA, Dvorkin M, Kulikov AS, Lesin VM, Nikolenko SI, Pham S, Prjibelski AD, Pyshkin AV, Sirotkin AV, Vyahhi N, Tesler G, Alekseyev MA, Pevzner PA 2012 SPAdes: a new genome assembly algorithm and its applications to single-cell sequencing. J Comput Biol 19:455–477. doi:10.1089/cmb.2012.0021. 22506599 PMC3342519

[10] Bosi E, Donati B, Galardini M, Brunetti S, Sagot MF, Lió P, Crescenzi P, Fani R, Fondi M 2015 Medusa: a multi-draft based scaffolder. Bioinformatics 31:2443–2451. doi:10.1093/bioinformatics/btv171. 25810435

[11] Monnet C, Loux V, Gibrat JF, Spinnler E, Barbe V, Vacherie B, Gavory F, Gourbeyre E, Siguier P, Chandler M, Elleuch R, Irlinger F, Vallaeys T 2010 The *Arthrobacter* *arilaitensis* Re117 genome sequence reveals its genetic adaptation to the surface of cheese. PLoS One 5:e15489. doi:10.1371/journal.pone.0015489. 21124797 PMC2991359

[12] Niewerth H, Schuldes J, Parschat K, Kiefer P, Vorholt JA, Daniel R, Fetzner S 2012 Complete genome sequence and metabolic potential of the quinaldine-degrading bacterium *Arthrobacter* sp. Rue61a. BMC Genomics 13:534. doi:10.1186/1471-2164-13-534. 23039946 PMC3534580

[13] Nakatsu CH, Barabote R, Thompson S, Bruce D, Detter C, Brettin T, Han C, Beasley F, Chen W, Konopka A, Xie G 2013 Complete genome sequence of *Arthrobacter* sp. strain FB24. Stand Genomic Sci 9:106–116. doi:10.4056/sigs.4438185. 24501649 PMC3910542

[14] Mongodin EF, Shapir N, Daugherty SC, DeBoy RT, Emerson JB, Shvartzbeyn A, Radune D, Vamathevan J, Riggs F, Grinberg V, Khouri H, Wackett LP, Nelson KE, Sadowsky MJ 2006 Secrets of soil survival revealed by the genome sequence of *Arthrobacter aurescens* TC1. PLoS Genet 2:e214. doi:10.1371/journal.pgen.0020214. 17194220 PMC1713258

[15] Angiuoli SV, Gussman A, Klimke W, Cochrane G, Field D, Garrity G, Kodira CD, Kyrpides N, Madupu R, Markowitz V, Tatusova T, Thomson N, White O 2008 Toward an online repository of Standard Operating Procedures (SOPs) for (meta)genomic annotation. OMICS 12:137–141. doi:10.1089/omi.2008.0017. 18416670 PMC3196215

[16] McArthur AG, Waglechner N, Nizam F, Yan A, Azad MA, Baylay AJ, Bhullar K, Canova MJ, De Pascale G, Ejim L, Kalan L, King AM, Koteva K, Morar M, Mulvey MR, O’Brien JS, Pawlowski AC, Piddock LJV, Spanogiannopoulos P, Sutherland AD, Tang I, Taylor PL, Thaker M, Wang W, Yan M, Yu T, Wright GD 2013 The Comprehensive Antibiotic Resistance Database. Antimicrob Agents Chemother 57:3348–3357. doi:10.1128/AAC.00419-13. 23650175 PMC3697360

[17] Weber T, Blin K, Duddela S, Krug D, Kim HU, Bruccoleri R, Lee SY, Fischbach MA, Müller R, Wohlleben W, Breitling R, Takano E, Medema MH 2015 antiSMASH 3.0–a comprehensive resource for the genome mining of biosynthetic gene clusters. Nucleic Acids Res 43:W237–W243. doi:10.1093/nar/gkv437. 25948579 PMC4489286

